# To the Editors of the Medical and Physical Journal

**Published:** 1807-06

**Authors:** 


### *To the Editors of the Medical and Physical Journal.*

Gentlemen,

It is with some diffidence I venture to offer a Critique upon the opinions and practice of a Gentleman, who, from his situation in a Naval Hospital, must possess peculiar advantages; but it appears to me that the case of James Burn was mistaken in the first instance. If a more correct idea had been formed of the nature of the accident, a different mode of treatment would perhaps have been thought necessary; the circumstances of the Case, which lead me to form this opinion, are,

1st. The violent pain the man felt at the time of the accident, "which was so severe that it rendered him totally unable to go on with his duty, and obliged him to retire," seems to me to have been occasioned by a rupture of the artery, particularly so as he felt something "snap" at the time.

2dly. Is it not unlikely that a *dilatation* of the artery could have become so considerable in so short a time as six weeks, "as to leave no doubt about the nature of the disease." In all cases of true aneurism, which I have seen or read of, the progress was much slower. If this was the real state of the case, the operation which was performed was very precarious, if not highly improper. The event of the operation proves, that the artery was supplied with blood below the ligature as freely as before the operation, as least this was the case twelve days after the operation; for there cannot be the least doubt but the blood came from the ruptured femoral artery, as no anastomozing branch, let it be ever so much enlarged, could pour out "four pounds of blood in half a minute."

On account of the situation of the wound, the ligature above the tendon of the triceps was too far removed from it to have any effect in suppressing hæmorrhage, when the anastomosing branches became enlarged; and on account of the free communication which is kept up between the branches in the leg, it became necessary that a second ligature should have been applied upon the lower portion of the artery.

From this view of the case, it appears that the proper operation would have been to secure the artery at the place where it was ruptured; this would not have been very difficult, as the extravasated blood always separates the artery from the surrounding parts, and upon turning out the coagula the wound in the artery commonly appears.

I perfectly agree with the gentleman in thinking that too much time was lost before any operation was performed. I have now had experience enough to determine that there is never any danger in securing the femoral artery; I never saw or read of a case, in which mortification to any extent took place in consequence. Two of three fatal cases which I have seen, proved so from ulceration taking place in a large tumour, after the operation had perfectly succeeded, as far as the obliteration of the vessel was concerned.

I believe a screw tourniquet will seldom succeed in suppressing bleeding so long after an operation for aneurism; a common stick tourniquet, by acting upon every muscular branch, will answer better, as the screw one only makes pressure at particular points.

Note, It is rather singular the gentleman never mentions the state in which the artery at the ham was found upon dissection.

I am, &c.

*April* 10, 1807. A MEMBER OF THE COLLEGE.

---

